# Staff well‐being: Is it time to rethink implications for work schedules?

**DOI:** 10.1111/medu.14991

**Published:** 2022-12-02

**Authors:** Gillian Nisbet

**Affiliations:** ^1^ Faculty of Medicine and Health The University of Sydney Sydney New South Wales Australia

## Abstract

As the world continues to navigate the impacts of COVID‐19, grappling with staff retention and emotional exhaustion, Nisbet shines a spotlight on healthcare professional well‐being.

Whenever we fly, the safety announcement directs passengers in case of emergency to first fit their own mask before attending to their child or other dependents. The logic is sound—a carer cannot help others if they are in danger themselves. In medicine, there is a strong professional commitment to altruism where the needs of the patient are placed above those of the medical practitioner. This principle is included as one of the arguments in the Accreditation Council for Graduate Medical Education's(ACGME) recently updated document supporting 24 hour shift lengths for PGY‐1 residents.^1^ However, how does this impact the health professional's, that is, the carer's own well‐being in such situations? Sephien and colleagues’^2^ systematic review investigating the evidence associated with resident doctor duty hour restrictions and its impact on resident and patient based outcomes is timely as we grapple with staff well‐being and workforce shortages, exacerbated by the COVID‐19 pandemic. The review identified that shorter shift lengths compared with longer shift lengths were associated with significantly less dissatisfaction with overall well‐being, less sleepiness and less emotional exhaustion. There was no association between reduced shift length and preventable adverse events or serious medical errors. These findings provide opportunities to reflect on implications for health care practice more generally, particularly around well‐being, workforce development and retention and continuing professional development (CPD) across the professions.

A carer cannot help others if they are in danger themselves.

Discussions around staff work environment and the effect on well‐being are not isolated to the medical profession; it is a whole of health care concern. For example, creating a healthy work environment is considered key to optimal patient outcomes and clinical excellence in nursing.^3^, ^4^ The importance of positive staff well‐being is increasingly being recognised by health care organisations, educational institutions, accreditation bodies and professional associations and is made explicit through the concept of the Quadruple Aim. The Quadruple Aim builds on from Berwick and colleague's Triple aim for optimising health system performance,^5^ adding a new dimension: improving the work life of health care practitioners.^6^, ^7^ Sephien et al.’s systematic review indirectly talks to this aspect of providing quality care through raising the importance of caring for ourselves.^2^

The importance of positive staff well‐being is increasingly being recognised by health care organisations, educational institutions, accreditation bodies and professional associations.

COVID‐19 has heightened concerns for staff well‐being, particularly the increasing risk of staff burnout.^8^ Burnout is defined as a “workplace syndrome characterised by high emotional exhaustion, high depersonalisation (e.g. cynicism) and a low sense of personal accomplishment from work”.^9^ While Sephien et al.’s review only found associations between shift length and one element of burnout on the Maslach Burnout Inventory,^10^ emotional exhaustion, all included studies were pre COVID‐19. There is now a plethora of COVID‐19 related studies highlighting the prevalence of burnout amongst health care workers across many countries and workplace settings.^8^, ^11^, ^12^

There is now a plethora of COVID‐19 related studies highlighting the prevalence of burnout amongst health care workers.

Poor staff well‐being and staff burnout negatively impact staff retention. Workforce shortage in health care related professions was a global problem prior to COVID‐19. Almost 3 years into COVID‐19, the problem has worsened.^13^ This can negatively impact staff morale and results in additional costs to the organisation in replacing staff and orientating new staff to the workplace. Recent studies have suggested that younger health care workers are at increased risk of burnout,^8^, ^14^ which would encompass the medical residents included in Sephien et al's review.^2^ Policy makers would be well‐advised to review all policies and practices, including work schedules, that could potentially influence a health care professional's decision to stay or leave their chosen profession.

It is important to consider the implications of well‐being for health professions' education and CPD. The tension already experienced by health care professionals juggling CPD within an already tight work schedule will only be compounded with feelings of exhaustion and poor well‐being. CPD is what may drop down the priority list. Indeed, many CPD programmes within health care organisations were put on hold during the heights of the pandemic (for safety as well as resource prioritisation). Yet CPD is intimately linked with providing high quality patient care. Without the capacity for CPD, patient care may ultimately be compromised.

Juggling CPD within an already tight work schedule will only be compounded with feelings of exhaustion and poor well‐being.

As previously mentioned, Sephien et al.’s review did not find an association between shift length and adverse events or serious medical errors.^2^ This is consistent with findings from an earlier review investigating medical practitioner burnout and patient outcomes.^15^ While medical practitioners reporting burnout perceived their clinical work quality to be compromised, clinical data did not support those perceptions.^15^ This may, in part, be due to the strong professional culture of putting the patient and the provision of high quality care above all else.^16^ But where does this leave our health care workers in the long term? Is this sustainable? Is there a tipping point?

The strong professional culture of putting the patient and the provision of high quality care above all else.

Improving work schedules of health care professionals is only one strategy for improving staff well‐being. It needs to be considered in the mix along with other organisational (systems‐based) interventions such as improving workflow management, improving communication, increasing teamwork and creating an environment where staff can easily access appropriate help and resources.^11^, ^13^ The need for action is made more urgent as we navigate this new world of living with COVID‐19.

Let us return to the opening scenario of the flight announcement requesting self‐care before assisting others. If only health care delivery was as simple as that! It is not. But there has to be a balance. There has been considerable investment in recent years to develop resources to assist individual health care practitioners. These, however, must also align with cultural and system change if we are to realise the final element of the Quadruple Aim.

## AUTHOR CONTRIBUTIONS

Gillian Nisbet is the sole author for this commentary.
